# Impact of pericardiectomy on exercise capacity and sleep of patients with chronic constrictive pericarditis

**DOI:** 10.1371/journal.pone.0223838

**Published:** 2019-10-11

**Authors:** Dirceu Thiago Pessoa de Melo, Flavia Baggio Nerbass, Ana Luiza Carrari Sayegh, Francis Ribeiro de Souza, Viviane Tiemi Hotta, Vera Maria Curi Salemi, Félix José Alvarez Ramires, Ricardo Ribeiro Dias, Geraldo Lorenzi-Filho, Charles Mady, Fábio Fernandes

**Affiliations:** 1 Cardiomyopathy Clinical Unit, Cardiology Division, Heart Institute (InCor), Hospital das Clínicas HCFMUSP, Faculdade de Medicina, Universidade de São Paulo, São Paulo, SP, Brazil; 2 Sleep Laboratory, Pulmonary Divison, Heart Institute (InCor), Hospital das Clínicas HCFMUSP, Faculdade de Medicina, Universidade de São Paulo, São Paulo, SP, Brazil; 3 Unit of Cardiovascular Rehabilitation and Exercise Physiology Cardiology Division, Heart Institute (InCor), Hospital das Clínicas HCFMUSP, Faculdade de Medicina, Universidade de São Paulo, São Paulo, SP, Brazil; 4 Heart Failure Unit, Cardiology Division, Heart Institute (InCor), Hospital das Clínicas HCFMUSP, Faculdade de Medicina, Universidade de São Paulo, São Paulo, SP, Brazil; 5 Unit of Cardiac Surgery, Cardiology Division, Heart Institute (InCor), Hospital das Clínicas HCFMUSP, Faculdade de Medicina, Universidade de São Paulo, São Paulo, SP, Brazil; Ospedale del Cuore G Pasquinucci Fondazione Toscana Gabriele Monasterio di Massa, ITALY

## Abstract

**Background:**

Heart failure is associated with exercise intolerance and sleep- disordered breathing; however, studies in patients with chronic constrictive pericarditis are scarce. The purpose of our study was to assess exercise capacity and sleep in patients with chronic constrictive pericarditis (CCP) undergoing a pericardiectomy.

**Methods:**

We studied consecutive patients scheduled for pericardiectomy due to symptomatic CCP. Were performed quality of life (Minnesota Living with Heart Failure Questionnaire—MLHFQ) and sleep questionnaires (Epworth, Pittsburgh Sleep Quality Index—PSQI), serum B-type natriuretic peptide (BNP), serum C-reactive protein, transthoracic echocardiography, cardiopulmonary exercise test and overnight polysomnography immediately before and six months after pericardiectomy.

**Results:**

Twenty-five patients (76% males, age: 45.5±13.8 years, body mass index: 24.9±3.7 kg/m^2^, left ventricular ejection fraction: 60±6%) with CCP (76% idiopathic, 12% tuberculosis) were studied. As compared to the preoperative period, pericardiectomy resulted in reduction in BNP (143 (83.5–209.5) vs 76 (40–117.5) pg/mL, p = 0.011), improvement in VO_2_ peak (18.7±5.6 vs. 25.2±6.3 mL/kg/min, p<0.001), quality of life (MLHFQ score 62 (43,5–77,5) vs. 18 (8,5–22), p<0,001) and sleep (PSQI score 7.8±4.1 vs. 4.7±3.7, p<0.001) and no significant change in sleep disordered breathing (apnea hypopnea index—AHI 15.6 (8.3–31.7) vs. 14.6 (5.75–29.9) events/h, p = 0.253).

**Conclusion:**

Patients with symptomatic CCP showed reduced exercise capacity and sleep-disordered breathing. After pericardiectomy, there was improvement in exercise capacity and neutral effect on sleep-disordered breathing.

## Introduction

Chronic constrictive pericarditis (CP) is the result of long standing inflammation of the pericardium that leads to fibrosis, lack of elasticity and ventricular systolic and diastolicdysfunction [1]. Typically, CCP patients show reduced exercise capacity and systemic congestion. Several mechanisms contribute to exercise intolerance in CCP, including diastolic dysfunction, myocardial atrophy and pulmonary hypertension [2]. Cardiopulmonary testing is the most useful tool to objectively assess the exercise capacity of patients with heart failure [3]. This test enables the assessment of prognosis, efficacy of treatment and selection for heart transplantation [4,5]. Moreover, cardiopulmonary testing plays an important role in prescribing exercises and rehabilitation programs.

Obstructive and central sleep apnea are common in patients with heart failure [6]. Both forms of sleep disordered breathing (SDB) are associated with fragmented sleep, intermittent hypoxia and neurohumoral disturbances that in turn may influence exercise capacity and survival [7,8,9]. The mecanisms linking sleep apnea and heart failure are not fully clarified, but seems to be associated to body fluid dynamics. In a study by Yumino et al, nocturnal rostral fluid shift from lower limbs to the neck correlated directly with the presence of obstructive and central sleep apnea in patients with heart failure with reduced ejection fraction (HFrEF) [10]. These findings open perspectives for studies investigating the effects of treatment of HF in sleep apnea.

Pericardiectomy remains the gold standard treatment for severely symptomatic patients with CCP. However, pericardiectomy has a high perioperative mortality, and heart failure symptoms do not improve in some patients [11,12]. The impact of pericardiectomy on the cardiopulmonary capacity and sleep in symptomatic patients with CCP has not been studied. Therefore, our goal was to test the hypothesis that pericardiectomy improves exercise capacity, sleep quality and SDB in patients with severe CCP.

## Methods

### Patient population

We prospectively studied 25 consecutive patients with CCP and indication of radical pericardiectomy between February 2011 and November 2015 in the Heart institute, São Paulo State University, a tertiary cardiology hospital. The diagnosis of CCP was based on clinical, echocardiographic and cardiac magnetic resonance imaging (MRI) criteria according to European Society of Cardiology guidelines [1]. Cardiac MRI data were collected from medical records and the exam was not part of the prospective protocol. All patients were evaluated by specialized group that decided the surgery based on the presence of heart failure symptoms (NYHA functional class II, III, IV). Coronary angiography was performed in those older than 40 years old. We performed the following evaluations before and six months after surgery: echocardiography, serum B-type natriuretic peptide (BNP) levels (Siemens, California, USA), cardiopulmonary exercise test, overnight complete nocturnal polysomnography, quality of life (Minnesota Living with Heart Failure) and sleep evaluation questionnaires (Epworth Sleepiness Scale, Pittsburgh Sleep Quality Index). Patients older than 70 years or with pulmonary disease were excluded from the study.

The etiology of CCP was defined according to a standardized investigation. Tuberculous CCP was confirmed by a pericardium biopsy as the occurrence of caseating granuloma or a positive test result of tubercle bacilli, as confirmed by polymerase chain reaction. Postsurgical constriction was defined as CCP after cardiac surgery. Constriction secondary to systemic inflammatory disease was diagnosed in patients with documented autoimmune disease. Idiopathic constriction was diagnosed when patients did not qualify for any of the previous groups. All patients provided signed informed consent and the study was approved by the institutional review board (CAPPESQ number: 202.007 –Comissão de Ética para Análise de Projetos de Pesquisa). This study complies with the Declaration of Helsinki.

### Echocardiography

The echocardiographic study was performed using a Sequoia 512 ultrasound device (Acuson, Mountain View, California, USA) with a 2.5 MHz transducer. All measurements were performed according to the American Society of Echocardiography guidelines [13]. A nasal respirometer was used for simultaneous recording of respiration. Examinations were performed by an experimental examiner blinded to other assessments of the protocol. Two-dimensional imaging was performed from parasternal, apical, and subcostal windows. The parasternal and apical views and M-mode recordings were used to detect the presence of respiratory ventricular septal motion. Apical views were also used to detect distortion of ventricular contours by CCP. The subcostal view was used to identify diameters of the inferior vena cava. Doppler information was obtained from apical, subcostal, right supraclavicular, and parasternal imaging windows. From the apical window, pulsed-wave Doppler recordings at the level of the mitral leaflet tips were used to measure early (E) and atrial (A) diastolic velocities, deceleration time of the E wave, and respiratory variation in the E velocity. Tissue Doppler assessment of mitral annular motion was used to record and compare medial and lateral early (e') relaxation diastolic velocities.

### Cardiopulmonary exercise test

Functional capacity was evaluated using a cardiopulmonary exercise test (CPT) according to American Heart Association guidelines [3]. The exercise test was performed on a treadmill (Ergoline ViaSprint 150P) with a modified Balke protocol consisting of velocities varying from 2 to 3.4 mph and ramp increments of 2% per minute. Oxygen (O_2_) and carbon dioxide (CO_2_) fractions were measured at each respiratory cycle. This evaluation was performed using a computerized system (SensorMedics, V_max_ Analyzer Assembly, Encore 29S). Individuals were considered to have reached their maximum when found at least one of the following parameters: respiratory exchange ratio >1.10; heart rate >95% of that predicted for age; extreme tiredness.

### Sleep study

All patients underwent overnight polysomnography as previously described (EMBLA, *Medical Devices*, *Broomfield*, *USA*) [14]. Apnea was defined by complete cessation or reduction of 90% of airflow for at least 10 seconds that was accompanied by oxyhemoglobin desaturation by at least 3%. Hypopnea was defined as a reduction of >50% in airflow for at least 10 seconds that was accompanied by oxyhemoglobin desaturation by at least 3%. Obstructive apnea was defined based on the presence of thoracic and abdominal efforts. Central apnea was defined in the absence of thoracic or abdominal efforts. Sleep apnea was defined as an AHI ≥15 events/h.

### Questionnaires

#### Minnesota Living with Heart Failure Questionnaire

The Minnesota Living with Heart Failure Questionnaire (Portuguese version) was used to evaluate patient quality of life and was administered as previously described [14]. It consists of 21 self-administered questions that address emotional and physical aspects of patients with heart failure. Each question should be answered on a scale of 0 to 5. Total score vary from 0 to 105. The higher the score, the worse the quality of life [15].

#### Epworth Sleepiness Scale

The Epworth Sleepiness Scale was used to evaluate subjective excessive daytime sleepiness [16]. Briefly, a score >10 points was considered to indicate excessive daytime sleepiness.

#### Pittsburgh Sleep Quality Index

Sleep quality was assessed using the Pittsburgh Sleep Quality Index as previously described [17]. A score ≥5 points was considered to indicate poor sleep quality.

### Statistical analysis

Statistical analysis was performed using SPSS 17.0 (SPSS Inc., Chicago, IL, USA). Quantitative variables were expressed as the means ± SD or median and interquartile range. Categorical data were summarized as frequencies and percentages. The preoperative and postoperative variables were compared using the paired T test (normal distribution) or the Wilcoxon test (non-normal distribution) and McNemar's test for categorical data. Correlations between variables were performed using the Spearman or Pearson method, when appropriate.

For the evaluation of the predictors of functional capacity improvement after pericardiectomy, we used multivariate analysis with predicted VO_2_ variation as a dependent variable (ΔVO_2_% = postoperative predicted VO_2_ peak—preoperative predicted VO_2_ peak). We initially selected baseline characteristics in the univariate analysis with p<0.10. Next, we performed a multivariate linear regression model with a stepwise selection process to identify independent predictors of VO_2_ peak improvement. A p-value of 0.05 was considered statistically significant.

## Results

### Patients

We evaluated 31 patients between February 2011 and November 2015. One patient died from cardiogenic shock before enrollment and two patients were excluded due to severe pulmonary disease. Twenty-eight were enrolled and three patients lost follow-up; 25 patients completed the study. Baseline characteristics of the patients included in the study are described in Table 1. The patients were predominantly middle-aged males. The predominant etiology was idiopathic, followed by tuberculosis. Most patients presented with preserved left ventricular ejection fraction (LVEF) with limiting symptoms and clinical signs of hypervolemia despite medical treatment. All patients underwent phrenic-to-phrenic pericardiectomy via median sternotomy, without extracorporeal circulation. The resection plane was extended to the diaphragmatic face of the heart. When possible, resection of visceral and parietal pericardium was performed. In three cases, the resection was considered incomplete due to the presence of extensive calcification and adhesion to the epicardium. In the other twenty-three cases pericardiectomy was considered complete. Two patients underwent concomitant coronary artery bypass grafting due to the presence of a significantly obstructive lesion in the left anterior descending artery and in the anomalous right coronary. The mean duration at the intensive care unit (ICU) was 3.4±3.3 days. The ICU duration of two patients was prolonged (>3 days) by the presence of cardiogenic shock, which was a secondary complication of pericardial decompression syndrome and was controlled after supportive measures with inotropes. After hospital discharge, which occurred on average on the 12th postoperative day, all patients had an ambulatory visit at 15, 90 and 180 days. During this time, two patients were readmitted to the hospital with decompensated heart failure. In one case, thoracic surgery was performed via median sternotomy to successfully drain a serous mediastinal collection with signs of right ventricular compression. In the second case, heart failure was controlled with adjustment of medical treatment. None of the patients entered a rehabilitation program after hospital discharge.

**Table 1:** Baseline characteristics of patients.

Variable	Value (n = 25)
Age (years)	45.5±13.8
Male sex, n (%)	19 (76)
BMI (kg/m²)	24.9±3.7
Neck circumference (cm)	37 (36–38.3)
Waist circumference (cm)	93.6±8.7
Etiology	
Idiopathic, n (%)	19 (76)
Tuberculosis, n (%)	3 (12)
Postoperative, n (%)	1 (4)
Systemic inflammatory disease, n (%)	2 (8)
Symptom time (months)	24 (12–36)
NYHA functional class III/IV, n (%)	14 (56)
Ascites, n (%)	18 (72)
Lower limb edema, n (%)	22 (88)
Cardiac resonance	
LVEF (%)	58±11
Pericardial thickening (mm)	7.2±3.6
Septal bounce, n (%)	23 (92)
Respiratory variation in Inferior vena cava, n (%)	23 (92)
Pericardial LGE	6 (24)
Myocardial LGE	2 (8)
Chest Radiograph—pericardial calcification, n (%)	11 (44)
Hypertension, n (%)	4 (16)
Diabetes, n (%)	2 (8)
Smoking, n (%)	5 (20)
Atrial fibrillation, n (%)	10 (40)
Furosemide (mg)	40 (30–80)
Spironolactone (mg)	25 (0–62.5)
BNP (pg/mL)	143 (83.5–209.5)
CRP (mg/dL)	5.4 (3–10)
Creatinine (mg/dL)	1 (1–1.2)
Hemoglobin (g/dL)	13.7±2

Data with normal distribution are presented as the mean ± standard deviation. Data with abnormal distribution are presented as the median and interquartile range. BMI: body mass index; NYHA: New York Heart Association; LVEF: left ventricular ejection fraction; LGE: late gadolinium enhancement; BNP: B-type natriuretic peptide; CRP: C-reactive protein

**Fig 1:**
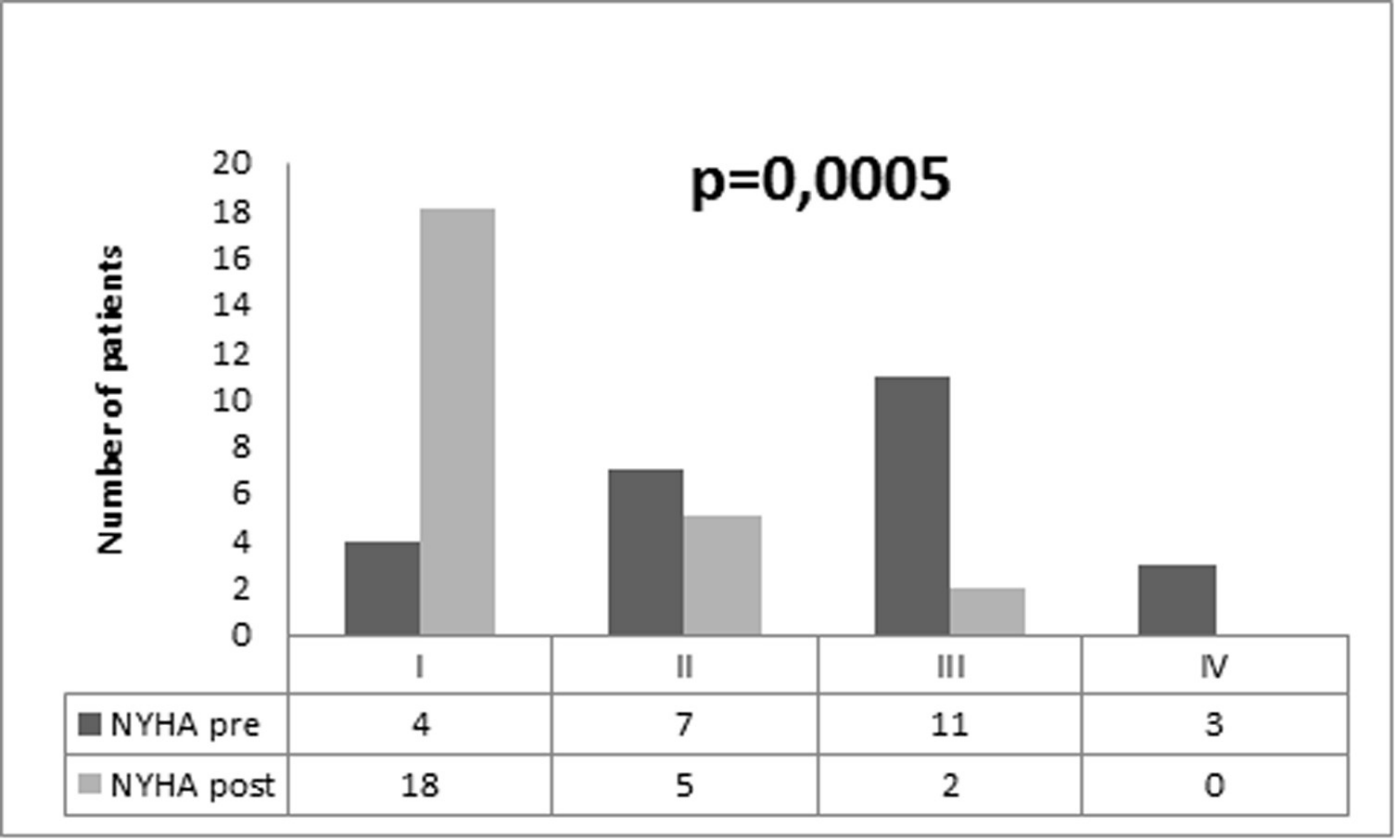
Changes in NYHA functional class six months after pericardiectomy.

The effects of pericardiectomy on clinical characteristics and quality of life after six months are listed in Table 2. Overall, after pericardiectomy there was an improvement in functional class and heart failure symptoms (Fig 1). Additionally, we observed a reduction in serum levels of BNP and C-reactive protein.

**Table 2:** Effect of pericardiectomy on quality of life, clinical and laboratory parameters.

Variable	Pre (n = 25)	Post (n = 25)	*p*
**MLHFQ score**	62 (43,5–77,5)	18 (8,5–22)	<0,001
**Anthropometric**			
Weight (kg)	73.9±14	74.2±16	0.722
BMI (kg/m²)	24.9±3.7	25.5±3.9	0.181
Waist circumference (cm)	93.6±8.7	89±8.7	**0.002**
**Clinical**			
Venous jugular distention, n (%)	22 (88)	10 (40)	**<0.001**
Ascites, n (%)	18 (72)	3 (12)	**<0.001**
Lower limb edema, n (%)	22 (88)	6 (24)	**<0.001**
**Laboratory**			
BNP (pg/mL)	143 (83.5–209.5)	76 (40–117.5)	**0.011**
CRP (mg/dL)	5.4 (3–10)	2.5 (1.8–4.5)	**0.014**
Hemoglobin (g/dL)	13.7±2	14.3±2.2	0.138
Creatinine (mg/dL)	1 (1–1.2)	0.9 (0.8–1)	**0.007**
**Drugs**			
Furosemide (mg)	56±50^a^	26.4±37.7^b^	**0.021**
Spironolactone (mg)	25 (0–62.5)^c^	0	**0.002**
Betablockers, n (%)	6 (24)	11 (44)	0.095

^a^ n = 2

^b^ n = 1

^c^ n = 15

Data with normal distribution are presented as the mean ± standard deviation. Data with abnormal distribution are presented as the median and interquartile range. BMI: body mass index; HR: heart rate; BP: blood pressure; BNP: B-type natriuretic peptide; MLHFQ: Minnesota Living with Heart Failure Questionnaire.

### Cardiopulmonary exercise test

The results of the cardiopulmonary test before and six months after surgery are summarized in Table 3. The test proved to be safe, without serious complications in all cases. The tests were considered effective once median respiratory exchange ratio was 1.1 at both times of the study. In one patient, the test was interrupted due to non-sustained ventricular tachycardia at the peak of the effort, with no signs of instability. Two patients had limiting symptoms at rest and did not undergo preoperative examination. In the postoperative period, one patient did not perform the test due to decompensated atrial flutter.

**Table 3:** Effect of pericardiectomy on functional capacity.

Variables	Pre (n = 23)	Post (n = 23)	*p*
Test duration (min)	11±3.1	11.9±1.9	0.166
Exercise time (min)	8.9±3.1	9.8±1.9	0.166
Treadmill speed (mph)	2.5 (2–2.5)	3 (2.5–3.3)	**0.001**
HR peak (bpm)	136.4±29.9	157.3±26.5	**0.002**
LA-VO_2_ (mL/kg/min)	13.1±3	17.7±5.5	**<0.001**
LA (%)*	70.7±11.3	70.5±9.3	0.953
VO_2_ peak (mL/kg/min)	18.7±5.6	25.2±6.3	**<0.001**
VO_2_ peak (%)*	61.7±18.9	83.6±24.7	**<0.001**
V_E_ (L/min)	50.4±13.8	61.1±19.7	**<0.001**
V_E_/VCO_2_*slope*	32.7±5.9	27.1±6,5	**0.001**
RER	1.1 (1.0–1.2)	1.1 (1.0–1.2)	**0.224**

* Percentage in relation to predicted for age and sex. Data with normal distribution are presented as the mean ± standard deviation. Data with abnormal distribution are presented as the median and interquartile range. LA-VO_2_: oxygen consumption at the lactate (anaerobic) threshold; LA: lactate (anaerobic) threshold; HR: heart rate; VO_2_: oxygen consumption; V_E_: pulmonary ventilation; RER: respiratory exchange ratio.

**Fig 2:**
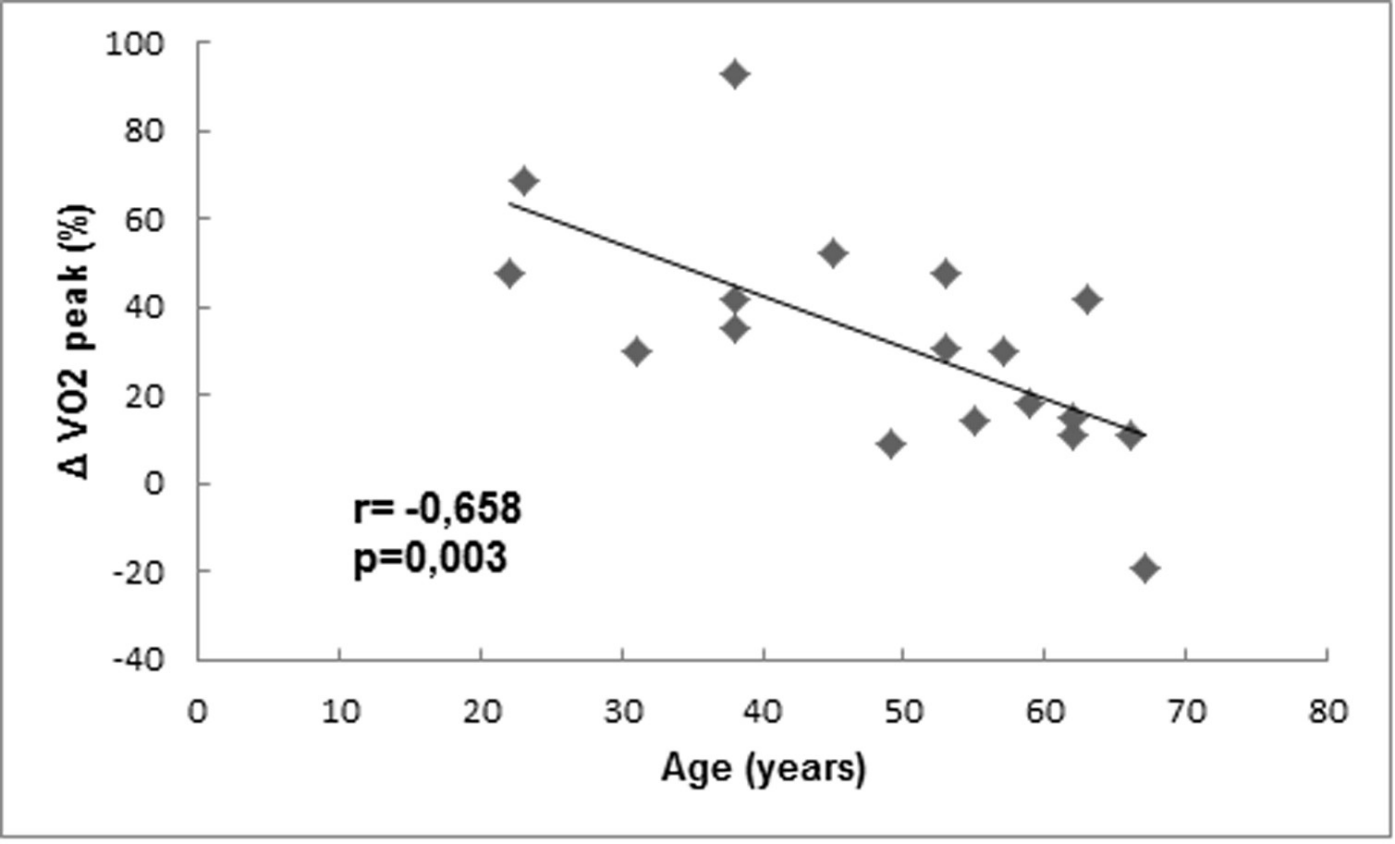
Correlation between VO_2_ peak variation and age. The final model of multivariate analysis is described by the formula Δ VO_2_ peak (%) = 89,5–1,17 x age.

Overall, patients presented improvement in cardiopulmonary capacity after surgical intervention. There was a significant increase in treadmill speed, anaerobic threshold and VO_2_ peak. In the preoperative period, there was a moderate inverse correlation between the VO_2_ peak predicted for age and the quality of life score (r = -0.432, p = 0.034). In univariate analysis, the following variables showed correlation (p<0,10) with VO_2_ peak variation: age, central apnea, Pittsburgh score, exercise time, anaerobic threshold, time with oxygen saturation <90% during sleep). Finally, in multivariate analysis age was the only independent predictor of VO_2_ peak variation (Fig 2).

### Sleep study

Sleep study results are summarized in Table 4. SDB (AHI≥15 events/hour) was diagnosed in 13 patients (52%) during the preoperative period. However, the respiratory events were predominantly hypopneas with no significant oxygen desaturation and no symptoms of excessive daytime sleepiness. Pericardiectomy resulted in no significant change in polysomnographic paramenters, including total sleep time, sleep efficiency, architecture and AHI. In contrast, sleep quality evaluated by PSQI improved significantly after surgery. In the preoperative period, there was a positive correlation between AHI and serum BNP levels (r = 0.418, p = 0.037).

**Table 4:** Effect of pericardiectomy on polysomnography and sleep questionnaires.

Variables	Pre (n = 25)	Post (n = 25)	*p*
Total sleep time (min)	350 (323–388.7)	368 (347.2–398.2)	0.199
Sleep efficiency (%)	91.7 (87.2–95.8)	90.5 (76.6–92.6)	0.086
S1 (%)	9.3 (6.3–16.2)	8.4 (5.8–13.6)	0.503
S2 (%)	55.4 (46.8–61.3)	48.5 (42.3–59.3)	0.475
S3 (%)	17.4±9.2	15±6.9	0.309
REM (%)	16±7.9	15.6±5.8	0.840
AHI (events/h)	15.6 (8.3–31.7)	14.6 (5.8–29.9)	0.253
Obstructive	0.8 (0–1.4)	0.5 (0–3.4)	0.217
Central	0.2 (0–1.4)	0.2 (0–0.7)	0.138
Mixed	0 (0–0.4)	0 (0–0.2)	0.610
Hypopnea	9.8 (6.35–20.1)	10.8 (4.9–16.6)	0.278
Mean oxygen saturation (%)	94.4±1.9	94.8±1.6	0.321
Minimal oxygen saturation (%)	85.6±4.7	85.5±4.6	0.689
Oxygen saturation <90% (min)	0.7(0–6.1)	0.9 (0–3.7)	0.862
**Sleep questionnaires**			
Epworth	7.6±3.6	6.5±4.3	0.239
Pittsburgh	7.8±4.1	4.7±3.7	**<0.001**

Data with normal distribution are presented as the mean ± standard deviation. Data with abnormal distribution are presented as the median and interquartile range. REM: rapid eye movement; AHI: apnea-hypopnea index.

### Echocardiography

Echocardiography data are summarized in Table 5. Before surgery, in general, patients presented with preserved ejection fraction, left atrial enlargement and inferior vena cava plethora. Respiratory variation of septal motion was identified in 17 patients. Tissue Doppler parameters revealed normal e' velocities with septal e'/lateral e' ratios that were compatible with a reversal annular flow in 17 patients (73.9%). The evaluation of the cardiac valves revealed moderate/severe tricuspid regurgitation in two cases.

**Table 5:** Effects of pericardiectomy on echocardiographic parameters.

Variables	Pre (n = 23)	Post (n = 23)	*p*
Aorta (mm)	30.2±3.10	31.6±4.5	0.090
Left atrium (mm)	45 (38–49)	42 (37–52)	0.676
Left atrium indexed volume (mL/m²)^a^	31.5 (24–38.7)	31 (26.4–38.6)	0.687
Diastolic volume LV (mL)	60 (51–64)	68 (52–81)	0.132
Systolic volume LV (mL)	22 (19–26)	25 (21–30)	0.112
LVEF Simpson (%)	60±6	60±4	0.661
Deceleration time (ms)	148.9±29.6	168.1±38.9	0.096
Mitral E velocity inspiration (cm/s)	65.7±22.7	70.7±22.4	0.291
Mitral E velocity expiration (cm/s)	84.1±27.7	86.5±24.4	0.625
Percent change mitral E velocity (%)	22.1±6.8	18.8±8.3	0.139
Respiratory septal motion, n (%)	17 (73.9)	11 (47.8)	0.065
Septal e' velocity (cm/s)	15.1±3.2	11.1±2.5	**<0.001**
Lateral e' velocity (cm/s)	14 (11–17)	12 (10–15)	0.303
Respiratory variation in inferior vena cava (%)	12±9	19.4±7.8	**<0.001**
PSAP (mmHg)	33±8.7	34.9±10.8	0.577

Data with normal distribution are presented as the mean ± standard deviation. Data with abnormal distribution are presented as the median and interquartile range. HR: heart rate; LVEF: left ventricle ejection fraction; PSAP: pulmonary artery systolic pressure.

^a^ n = 18.

## Discussion

We observed in a cohort of patients with symptomatic CCP improvement of symptoms, functional capacity, quality of life and sleep after pericardiectomy. In contrast we did not observe a significant impact of pericardiectomy on SDB.

Our study was the first to prospectively evaluate the cardiopulmonary exercise test in consecutive patients with CCP. In our sample, the patients presented a significant reduction in exercise capacity with VO_2_peak 18.7 ml/kg/min. After pericardiectomy, we observed an improvement in predicted VO_2_ peak, anaerobic threshold and VE/VCO2 slope. Moreover, the test was safe, and our baseline test results are similar to those from studies of patients with heart failure with preserved ejection fraction (HEFpEF) of other etiologies [18]. In ALDO-HF, a study that tested the effect of spironolactone on functional capacity of 422 patients with HEFpEF, mean baseline VO_2_ peak was 16.4 ml/min/kg. In a study of 156 patients with HEFpEF secondary to hypertrophic cardiomyopathy, meanVO_2_ peak was 26 ml/kg/min and independently associated with clinical outcomes [19]. Later, Yan et al. revealed that the serum BNP and V_E_/VCO_2_slope were strong predictors of mortality in a cohort of 224 patients with HEFpEF [20]. Based on these findings, we speculate that variables of the cardiopulmonary test might contribute prognostic information for patients with CCP. Also, the finding of age as an independent predictor of improvement in functional capacity is relevant since it suggests that benefits of pericardiectomy appear to be lower in older patients.

The association between sleep apnea and HFpEF is described in literature. In a prospective study of 244 patients with a mean age of 66 years and HFpEF, Bitter et al showed sleep apnea in 69% of the cases (AHI ≥5 events/hour). In addition, patients with apnea had higher levels of NT-pro-BNP, lower exercise capacity and worse diastolic function than patients without sleep apnea [21]. Of note, in this study the prevalence of hypertension, diabetes and overweight was high and patients with CCP were excluded. On the other hand, our sample consisted of relatively young, non-obese patients with few comorbidities and moderate/ severe apnea in 52% of the cases. Also, BNP showed a positive correlation with AHI. Thus, our hypothesis that sleep apnea is a result of hemodynamic (low cardiac output, edema) and neurohumoral changesof heart failure due to CCP is plausible. It should be noted, however, that esophageal transducer was not available to characterize the type of hypopnea (obstructive or central).

Considering the impact on cardiorespiratory function observed after pericardiectomy, the neutral effect on sleep apnea is intriguing. In a study by Gabor, resynchronization therapy reduced sleep apnea in patients with systolic heart failure [22]. Later, Bucca et al. demonstrated that treatment with loop diuretics reduced obstructive sleep apnea (AHI from 75 to 57 events/hour, p<0.001) in patients with HFpEF. [23]. Certain hypotheses may explain our results. First, despite the improvement in cardiopulmonary capacity, there was no complete normalization of cardiac hemodynamics. Second, although there was no statistically significant difference, patients with sleep apnea tended to be older. Of note, the improvement in sleep quality should be interpreted cautiously once there was a neutral effect on Epworth scale and sleep architecture. In our view, changes in Pittsburgh scale may be due to improvements in HF symptoms and quality of life.

Echocardiography showed a reduction in septal mitral e’ velocities reflecting a relief of lateral pericardial constraint and reduction in exaggerated longitudinal deformation of ventricles. These hemodynamic improvements may explain the changes in cardiopulmonary parameters. However, might not have been sufficient to modify sleep apnea events. Also, in our study the mean preoperative septal e’ (15 cm/s) was higher than published works (9–12 cm/s) [24,25,26]. This may be explained by the predominance of idiopathic cases (and absence of concomitant cardiomyopathy leading to e’ reduction) and more severe constriction. Despite clinical improvement, respiratory variation of inferior vena cava remained reduced (<50%) after pericardiectomy, indicating persistence of venous congestion. Indeed, in 1999 Senni et al. demonstrated abnormal ventricular filling in 59.5% of cases in a mean period of three months after pericardiectomy [27]. There are some hypotheses for these findings. First, in patients with prolonged symptoms and the presence of calcification, resection of the pericardium may not be complete [28]. Second, extension of the pericardial inflammatory process to the myocardium may result in myocardial damage and fibrosis, with consequent impaired filling [29] Finally, six months may not have been long enough to reach full effect of pericardiectomy on cardiac hemodynamics.

In our view, the study of objective measures of cardiorespiratory capacity in patients with chronic constrictive pericarditis is relevant because it opens perspectives for new studies that improve patient selection, risk stratification and evaluation of pericardiectomy results. Also, the mechanism by which CP patients develop sleep disordered breathing it’s not fully clarified and requires further investigation.

### Limitations

Our study has some limitations. The samples consisted of young patients with a predominance of idiopathic etiology in a tertiary cardiology center, which may limit the external validity of the results. In addition, the sample size of 25 patients limited the power of the study and invasive hemodynamic assessment of cardiac pressures was not performed. It should be noted, however, that this disease is rare and poorly diagnosed and that prospective studies are scarce in the literature.

## Conclusions

Patients with symptomatic CCP showed improvement in cardiopulmonary capacity, quality of life and sleep six months after pericardiectomy. Sleep apnea was frequent and did not show significant changes after pericardiectomy.

## Supporting information

S1 File Raw clinical data. (XLSX)

S2 File Raw data cardiopulmonary exercise testing. (XLSX)

S3 File Raw data Sleep study. (XLSX)

S4 File Raw data echocardiography. (XLSX)
